# The Effect of Benson Relaxation Technique on Depression, Anxiety, and Stress of Jordanian Patients Diagnosed with Multiple Sclerosis: A Cross-Sectional Study

**DOI:** 10.1155/2021/8300497

**Published:** 2021-10-13

**Authors:** Ahmad Rajeh Saifan, Mohannad Eid Aburuz, Enas A. Dhaher, Abdallah Rayyan, Mira Al Jaberi, Rami Masa'Deh

**Affiliations:** ^1^Nursing Department, Applied Science Private University, Amman, Jordan Postal Code: 11931, P.O. Box: 166; ^2^Nursing Department, Sharjah University, Sharjah, UAE; ^3^School of Medicine, University of Jordan, Amman, Jordan; ^4^Psychiatric Mental Health, Nursing Department, Applied Science Private University, Amman, Jordan

## Abstract

Depression, anxiety, and stress (DAS) are common symptoms of multiple sclerosis (MS) patients and are highly correlated with poor quality of life. Managing DAS among such patients can improve their quality of life (QoL), empowering them with improved autonomy, self-care, independency, and ability to perform daily activities. This study is aimed at examining the effectiveness of the Benson Relaxation Technique (BRT) on reducing DAS among patients diagnosed with MS in Jordan. This quasiexperimental study of 105 Jordanian patients diagnosed with multiple sclerosis tested an intervention group (60 patients) who received BRT and a control group (45 patients) who received normal treatment. Data were collected from January 2021 to April 2021, using the Arabic version of the Depression Anxiety Stress Scale (DASS21). The intervention group was instructed to perform the BRT two times a day for 10 minutes at home for eight weeks at two specific times, with 7-8-hour intervals between each episode. STROBE guidelines were followed in reporting the review. At the baseline comparison, there was no statistical difference between the interventional and control groups with regard to DAS. The levels of DAS between the two groups after three months of the last sessions of the intervention (postintervention) were compared. The results showed that the intervention group had significantly lower levels of DAS compared to the control group. The levels of the DAS were significantly lower for the intervention group postintervention. Adding relaxation techniques to the therapeutic routine is a cost-effective complementary treatment to decrease DAS among MS patients and improve their QoL. *Relevance to Practice*. This study provides a baseline of data that could facilitate further investigations in the future to improve the quality of services delivered to such patients and thus their QoL and satisfaction.

## 1. Introduction

Multiple sclerosis (MS) is a chronic disease affecting a patient's central nervous system. Epidemiological data on MS prevalence indicates that approximately 2.8 million people live with MS worldwide, which is equal to 35.9 per 100,000 population [1]. In the Middle East and North Africa (MENA), the prevalence of MS is much higher than the global average and is equal to 51.52/100,000 [2]. In Jordan, the average prevalence is relatively lower at 31 per 100,000 population, although the rate reported in the capital city (Amman) is 39 per 100,000 population [3]. Globally and in Jordan, females are twice as likely to be diagnosed with MS compared to males, with an average age of diagnosis of 31 years [1].

Symptoms of MS can affect the physical, cognitive, and emotional status of patients [4–6]. The most common physical symptoms reported in the literature include pain, fatigue, tingling sensation, weak bladder control, visual problems, vocal changes, difficulty breathing, muscle weakness, weak bones, and gastrointestinal dysfunction [4]. Moreover, cognitive impairment such as deficits affecting recent memory, attention span, and problems in learning new materials affect around two-thirds of patients [7]. Furthermore, DAS were the most common emotional changes reported by MS patients (MSPs) [8]. A meta-analysis found that emotional symptoms were prevalent in MSPs in almost a third of reviewed studies [9]. In Oman, depressive symptoms were found to affect almost half of the patients, and anxiety symptoms were reported in more than a third of MSPs [10]. Investigating this further, symptoms of depression and anxiety were investigated in 80 MSPs in UAE, and the results showed that major depression was reported by 17% of patients and anxiety by 20% [11]. In addition to depression and anxiety, stress was examined in 87 MSPs in Iran. The study showed that 47% of the patients had moderate depression, almost 40% had moderate anxiety, and 45% had moderate stress [12].

Emotional symptoms such as DAS are highly correlated with poor quality of life (QoL) among MSPs [13]. The same issue was reported in a review of 106 articles focused on the relationship between the emotional symptoms of MSPs and their QoL. The review reported negative impacts of DAS on MSPs' QoL [14]. It seems that DAS are very prevalent in MSPs and were found to affect their QoL. Therefore, managing DAS of those patients might improve their QoL, which in turn could give them a sense of control and independency and improve their self-efficacy and enjoyment in activities of daily living [14, 15].

Previous literature applied different methods aimed at minimizing the emotional impact of MS on patients [16, 17]. Relaxation techniques and stress management have been used to decrease DAS levels in MSPs [18]. For example, stress management interventions were found to be effective in reducing stress in eight randomized controlled trials involving 568 adults with MS [16]. Moreover, relaxation techniques as stress management tools were found to improve the overall QoL for MSPs [19]. Benson Relaxation Technique (BRT) was first introduced in 1975 and is considered one of the most effective relaxation techniques used to decrease DAS [20]. BRT was previously used in patients with chronic renal failure undergoing haemodialysis and found to be effective in reducing DAS [21]. One study conducted in Iran including women with MS reported that BRT was effective in reducing physical as well as emotional symptoms [22].

Although BRT seems to be effective in reducing the emotional symptoms of patients with chronic diseases, the effect of BRT on DAS symptoms has never been studied among MSPs in Jordan. Therefore, this study is aimed at examining the effectiveness of BRT on reducing DAS among patients diagnosed with MS in Jordan. The study answered the following research question:

- Does the Benson Relaxation Technique reduce the depression, anxiety, and stress of patients diagnosed with MS?

## 2. Materials and Methods

### 2.1. Design

This is a pre-post quasiexperimental study, involving two groups of MSPs: the interventional and control groups. The control group acted as a placebo group. This review is reported according to the Strengthening the Reporting of Observational studies in Epidemiology (STROBE) (see Supplementary File 1).

### 2.2. Setting

The study involved 11 neurological clinics in Amman: six are operated by the Ministry of Health, and five are in private hospitals.

### 2.3. Population, Sampling, and Sample Size

The study population included all patients diagnosed with MS in Jordan. Nonrandom convenience sampling technique was used to recruit patients. The inclusion criteria were (a) having a confirmed medical diagnosis of MS by a neurologist and (b) being able to understand, read, and write Arabic. Exclusion criteria were (a) having a psychotic disorder (as documented in their medical file), (b) receiving psychotherapy during the last six months, and (c) attending any intervention targeting psychological mental health during the previous six months. In the intervention group, those who were absent for any of the Benson interventional sessions were excluded from the study. Data were collected from June 2020 to December 2020.

The sample size was calculated using G power software [23]. Taking into consideration the statistical tests used in this study (i.e., independent *t*-test and paired *t*-test) and assuming a power of 80%, an *α* level of 0.05, and medium effect size, a total of 102 patients was deemed sufficient to detect any statistically significant difference between the two groups according to their demographics and was enough to detect the effect of BRT on the DAS of patients.

Of 145 patients approached and invited to voluntarily participate, 132 consented, representing 91%. Of those who consented, 105 completed and returned the questionnaire, representing a final response rate of 80%. They were divided into two groups, all of whom attended the two phases of the study: 60 patients received the intervention (i.e., the intervention group) and 45 had treatment as usual (i.e., the control group).

### 2.4. Instruments

#### 2.4.1. Sociodemographic Characteristics

The following information was collected for sociodemographic characteristics: age, gender, level of education, employment status, financial status, marital status, duration of disease, and frequency of relapse during the previous two years.

#### 2.4.2. Arabic Version of DASS21

The Depression Anxiety Stress Scale (DASS21) is a set of self-reported items used to measure the intensity of DAS over the week prior to administration. DASS21 is the short version of the basic 42-item questionnaire DAS S-42, developed by Lovibond and Lovibond [24]. It consists of three seven-item scales (a total of 21 items): depression scale (DS), anxiety scale (AS), and stress scale (SS) [24]. Items are measured through a four-point Likert scale from 0 (did not apply to me at all) to 3 (applied to me very much, or most of the time). Items in each scale are summed and doubled to be equivalent to the basic DAS S-42 scale, whereby higher scores indicate higher levels of DAS. A score between 0 and 4, 0 and 3, and 0 and 7 is considered normal DAS, respectively. A score between 5 and 6, 4 and 5, and 8 and 9 is considered mild DAS, respectively. A score between 7 and 10, 6 and 7, and 10 and 12 is considered moderate DAS, respectively. A score between 11 and 13, 8 and 9, and 13 and 16 is considered severe DAS, respectively. A score above 13, 9, and 16 is considered extremely severe DAS, respectively [24].

The reliability of DASS21 was measured using Cronbach's alpha and was reported to be good; it was 0.91 for the depression scale, 0.80 for the anxiety scale, and 0.84 for the stress scale [25]. The reliability of the Arabic version of DASS21 was measured using Cronbach's alpha coefficients and was found to be 0.76 for depression, 0.75 for anxiety, and 0.77 for stress [26]. In this study, the reliability of DASS21 was 0.83 for depression, 0.79 for anxiety, and 0.81 for stress.

#### 2.4.3. Benson Relaxation Technique (BRT)

BRT was given to the intervention group by one of the research team (interventionist) who is qualified in relaxation techniques. Participants in this group received two sessions of the Benson technique; one hour for each session in a warm quite room with efficient light, temperature, and ventilation was provided by the selected hospitals/clinics, and the technique was repeated twice. Participants then performed it in the presence of the interventionist, who provided feedback by evaluating participants' skills in performing the technique and ensured that they had acquired sufficient skills to do it alone. A video of BRT was displayed to participants during the sessions. Moreover, a CD of the Benson relaxation technique was provided with handout copies about the performance of BRT, so participants could watch the video and read the instructions when needed. Participants were instructed to perform the BRT as follows:

1. Sit quietly in a comfortable position
2. Close your eyes
3. Deeply relax all your muscles, beginning at your feet, progressing up to your face, and keep them deeply relaxed
4. Breath through your nose. Become aware of your breathing. As you breathe out, say the word “one” silently to yourself. Do deep breathing; inhale for three seconds, pause three seconds, and exhale over a three-second period
5. Continue for 10 minutes. You may open your eyes to check the time but do not use an alarm
6. When you finish, sit quietly for several minutes at first with closed eyes and later with opened eyes
7. Do not worry about whether you are successful in achieving a deep level of relaxation. Maintain a passive attitude and permit relaxation to occur at its own pace. When distracting thoughts occur, ignore them and continue repeating “one.” With practice, the response should come with little effort. Practice the technique twice a day, but not within two hours after any meal, as the digestive processes seem to interfere with the elicitation of anticipated changes

The intervention group was instructed to perform the BRT two times a day for 10 minutes at home for eight weeks at two specific times, 7-8-hour intervals between each episode. It was emphasized to participants that they should endeavour not to forget to perform the technique. However, if they forget to do it they were asked to do it as soon as possible.

There was a self-reported Performance Record Form, which was filled out daily by the participants to ensure their compliance with the BRT. Every week, the interventionist met the participants to encourage them to perform the technique, and at the same time, their Performance Record Form was collected. Moreover, to ensure that the participants underwent the intervention appropriately, the participants performed the technique again in the presence of the interventionist.

The interventionist established a WhatsApp group for all the participants and sent them the BRT video twice a day at specific times, to remind them to perform the technique. Furthermore, an identification card with the interventionist's contact information was given to the participants, and they were asked to contact the interventionist if they had any questions regarding the technique.

### 2.5. Ethical Considerations

Before starting with the data collection process, ethical approvals were gained from the Institutional Review Board of the Applied Science Private University and the Jordanian Ministry of Health to collect data from governmental hospitals. These were also presented to the private sector, and access was gained to all hospitals/clinics involved in the study. Participants' rights, such as voluntary participation, privacy, confidentiality, and the right to withdraw at any time without giving a reason, were guaranteed and explained to all participants. Participants were informed that all collected data were securely stored (i.e., in locked filing cabinets and password-protected computers), accessible only to the research team.

### 2.6. Data Collection Process

An invitation letter and an information sheet were sent to potential participants. The researchers' contact details were provided on the information sheet for those who wanted to participate or who had any enquiries about the study. An informed consent form was signed by those who agreed to participate. There was an option in the information sheet and the consent form to determine the patient's willingness to attend the intervention. Those who were willing to attend the program became the intervention group, and those who were unwilling to attend the program became the control. The participants then filled out the sociodemographic questionnaire and the DASS21 before the implementation of the program, and primary analysis was done. The course was then implemented over eight weeks. After that, participants were given three months before the second round of data collection was conducted, and DASS21 was filled out again.

### 2.7. Data Analysis Process

Data were analysed using SPSS version 25 [27]. All numbers in the results were rounded up to the closest two decimal points. Alpha was set as 0.05; therefore, any *p* value below 0.05 was considered significant. Descriptive statistics were used to describe the sociodemographic characteristics. Depending on the level of measurement of the variable, analysis tests were conducted. Due to the fact that DASS21 is a continuous dependent variable and there were two groups included in the study over two periods of time, chi square and *t*-test are the most suitable analysis techniques attending to the aim of the study. Chi square and independent *t*-test were conducted to compare the control group with the intervention group. A paired *t*-test was conducted to check any differences in DASS21 before and after implementing the course.

## 3. Results

Table 1 presents the sociodemographic characteristics of the sample based on the intervention and control groups. Depending on the level of the measurement of the variables, independent samples *t*-test and chi square were used to examine any difference between the intervention and control groups. Results showed that there were no statistically significant differences in any of these characteristics between the two groups. Both groups were young, with a mean age of 33 years. Nearly two-thirds of the sample were females, and half of them were married. The majority of the sample had a bachelor's degree and were employed. Exactly half of the intervention group was single, and 32.4 of the patients reported one time of relapse in the last two years. On the other hand, the highest percentage for the duration of the disease for both groups was between 1 and 2 years (Table 1).

To check the effect of the Benson relaxation intervention on MSPs' levels of DAS, four steps were implemented. Firstly, the levels of the outcome variables between the two groups at baseline prior to initiation of the intervention (i.e., preintervention) were compared using independent samples *t*-test, to make sure that there were no differences and to avoid bias. The results showed that there were no differences between the two groups (Table 2). The levels of the outcome variables between the two groups were then compared after three months of the last sessions of the intervention (postintervention) using an independent samples *t*-test. The results showed that the intervention group had significantly lower levels of DAS compared to the control group (Table 2).

A paired *t*-test compared the preintervention and postintervention levels of the outcome variables for the intervention and control groups. The results showed that there were significant reductions in DAS levels postintervention for the intervention group and no significant reductions for the control group (Table 3).

## 4. Discussion

The purpose of this study was to check the impact of implementing BRT on DAS among MSPs. In the current study, patients reported high DAS levels before the intervention. It is important to remember that MS is a complex neurological autoimmune disorder that progressively deteriorates the central nervous system [28]. This disorder causes several mental and physical symptoms, such as muscle weakness, balance problems, abnormal walking mechanics, spasticity, fatigue, cognitive impairment, and depression [29]. Such outcomes are believed to cause or exacerbate the increased levels of DAS among MSPs, as secondary outcomes of the direct impacts of MS, and the burdens the illness imposes on individuals' mental and physical health.

The results showed that there were no statistically significant differences in sociodemographic characteristics in relation to DAS between the intervention group and the control groups before implementing the intervention, making the comparisons between the groups more valid. However, when comparing the postintervention and preintervention data, it was found that the intervention group had significantly lower levels of DAS compared to the control group, concluding that BRT was effective in reducing DAS among patients diagnosed with MS. These results are in agreement with several studies that indicated the role of BRT in reducing DAS in patients diagnosed with MS [22, 30, 31]. In the same line, several studies reported significant reductions in levels of DAS when implementing BRT [30, 32–35]. BRT is known to be highly beneficial in reducing the activity of the autonomic nervous system, improving the balance between the anterior and posterior hypothalamus, reducing sympathetic activity and catecholamine release, relieving muscle tension, reducing blood pressure and heart rate, and regulating breathing [36–39]. All these impacts may explain the significant influence of BRT on reducing participants' DAS levels.

Similarly, a clinical trial conducted in Iran among 60 MSPs to determine the effect of relaxation (Jacobson and Benson techniques) on DAS with equal numbers of experimental and control groups found that the intervention group receiving relaxation techniques exhibited significantly decreased DAS scores after the intervention, while the control group receiving routine care had no significant decrease [18].

Moreover, a study of 60 MSPs to determine the effects of BRT on MSPs' general health assigned patients equally to experimental and control groups and found that the mean scores of patients' general health scores before and after the intervention improved significantly in the experimental group but not in the control group [30]. Further investigations of general health subscale scores of anxiety and depression revealed that the mean scores were significantly reduced after the intervention in each subscale, whereby lower scores indicate a higher level of general health, with no significant results reported in the control group. This study also showed that the impacts of BRT go beyond its effect on mental health in MSPs, and it can also be effective for improving MSPs' physical symptoms and social functioning [30].

Additionally, Mirhosseini et al. [40] investigated the effect of BRT on fatigue among MSPs and found that BRT exercises significantly reduced the average fatigue severity, which may contribute to decreased DAS, and thus improved QoL. These positive outcomes in the intervention group could be attributed to the fact that BRT improves parasympathetic activity and calmness, reducing levels of neurotransmission through the body and negative disease impacts [41, 42].

Other relaxation techniques were found to improve the QoL of patients diagnosed with MS. An earlier study suggested that exercise training programs such as yoga and aquatic exercises should be integrated into the standard treatment of MS, as they have been shown to reduce fatigue, depression, and paraesthesia significantly in exercise groups compared to nonexercise control groups. In fact, those who did not exercise were 35 times more likely to report moderate to severe depression than those who did such exercises [43]. Similarly, it was reported that exercising programs led to improvements not only in depression but also in the objective and subjective sleep of MSPs [44]. This would undoubtedly have a significant effect on well-being and could be attributable to the inducing of a “relaxation response,” defined by Benson et al. as a physiological and homeostatic state that counteracts stress triggers [45]. More significantly, it was reported that practicing relaxation techniques for 30 minutes daily for six weeks reduced fatigue and improved sleep quality among MSPs [46]. BRT was also found to be effective in reducing pain severity, anxiety, stress, and fatigue and improving self-esteem and QoL for patients with various conditions, including heart failure, women after caesarean section, and patients undergoing haemodialysis [33, 35, 36, 47–49].

Previous studies have shown that BRT is more effective in managing DAS than comparable relaxation techniques [33, 50–52]. It is a simple, safe, effective, inexpensive, and easy-to-learn technique, which does not require any special equipment, resources, or skills [21, 30, 36, 51]. All these factors demonstrate the superiority of this technique over complementary therapies for managing DAS.

## 5. Conclusions and Implications

BRT is a behavioural method designed to cope with DAS. Improving the levels of DAS is extremely important for the treatment of MSPs, as these conditions often present with various other comorbidities that can affect patients' QoL. The implications of this study include the importance of adding BRT to therapeutic routine treatments. This technique is a useful and cost-effective complementary therapy to alleviate MSPs' DAS. Since the effect of BRT on DAS symptoms has never been studied among MSPs in Jordan, this study provides a baseline of data that could facilitate further investigations in the future to improve the quality of services delivered to such patients and thus their QoL and satisfaction.

## 6. Study Limitations

The major limitation of this study was the inclusion of only one city in Jordan; it would have been more representative of the national picture to include participants from other cities. However, the capital includes more than one-third of the whole Jordanian population, making it generally representative in itself, and MS prevalence is slightly higher than the national average in Amman, as mentioned previously. Furthermore, MSPs come from all Jordanian cities to seek health services in this city. A more fundamental limitation was the relatively small sample size used in this current study as a result of convenience sampling. While adequate for the purposes of this research, the inclusion of more participants would have strengthened the conclusions drawn from the results. This study implements a quaziexperimental design; a randomized control trial may add more and can better predict the effect of the intervention.

## 7. Relevance to Clinical Practice

- Improving the levels of depression, anxiety, and stress (DAS) is extremely important for the treatment of multiple sclerosis patients (MSPs), as these conditions often present with various other comorbidities that can affect patients' QoL
- This study highlights the importance of adding the Benson Relaxation Technique (BRT) to therapeutic routine treatments. This technique is a useful and cost-effective complementary therapy to alleviate MSPs' DAS
- The effect of BRT on DAS symptoms has never been studied among MSPs in Jordan
- This study provides a baseline of data that could facilitate further investigations in the future to improve the quality of services delivered to such patients and thus their QoL and satisfaction

## Figures and Tables

**Table 1:** Sociodemographic characteristics of the sample (*N* = 105).

Character	Total sample	Intervention (*n* = 60)	Control (*n* = 45)
*Age*	33.11 ± 5.84	33.18 ± 5.65	33.02 ± 6.15
*Gender*			
Male	41 (39)	23 (38.3)	18 (40)
Female	64 (61)	37 (61.7)	27 (60)
*Marital status*			
Single	53 (50.5)	30 (50)	23 (51.1)
Married	47 (44.8)	27 (45)	20 (44.4)
Divorced	5 (4.8)	3 (5)	2 (1.9)
*Education*			
Baccalaureate	92 (87.6)	52 (86.7)	40 (88.9)
Master	13 (12.4)	8 (13.3)	5 (11.1)
*Financial status*			
Comfortable	54 (51.4)	31 (51.7)	23 (51.1)
Tight	51 (48.6)	29 (48.3)	22 (48.9)
*Employment*			
Employed	81 (77.1)	46 (76.7)	35 (77.8)
Unemployed	24 (22.9)	14 (23.3)	10 (22.2)
*Duration of disease*			
Less than one year	14 (13.3)	8 (13.3)	6 (13.3)
Between 1 and 2 years	41 (39)	23 (38.3)	18 (40)
Between 2 and 3 years	36 (34.3)	21 (35)	15 (33.3)
More than 3 years	14 (13.3)	8 (13.3)	6 (13.3)
*Relapse for the last two years*	1.28 ± 1.14	1.28 ± 1.12	1.27 ± 1.18
Zero	33 (31.4)	18 (30)	15 (33.3)
One time	34 (32.4)	20 (33.3)	14 (31.1)
Two times	14 (13.3)	9 (15)	5 (11.1)
Three times	24 (22.9)	13 (21.7)	11 (24.4)

Values are described as *n* (%) or *M* ± SD.

**Table 2:** Independent samples *t*-tests between the intervention and control groups pre- and postintervention.

Outcome variables	Group	*M* ± SD	*t*	*p* value
*Preintervention*				
Depression	Intervention control	17.87 ± 2.63 17.88 ± 1.75	-0.05	NS
Anxiety	Intervention control	17.67 ± 2.75 17.84 ± 2.06	-0.36	NS
Stress	Intervention control	18.58 ± 2.55 18.33 ± 1.51	0.59	NS
*Postintervention*				
Depression	Intervention control	12.45 ± 2.40 17.82 ± 1.98	-12.23	<0.001
Anxiety	Intervention control	12.68 ± 3.03 17.76 ± 2.07	-9.67	<0.001
Stress	Intervention control	16.87 ± 2.42 18.18 ± 1.56	-3.17	<0.01

NS: not significant.

**Table 3:** Paired samples *t*-tests between baseline and follow-up measurements for the intervention and control groups.

Outcome	Preintervention *M* ± SD	Postintervention *M* ± SD	*T*	*p* value
*Intervention group*				
Depression	17.87 ± 2.63	12.45 ± 2.4	15.29	<0.001
Anxiety	17.67 ± 2.75	12.68 ± 3.03	15.31	<0.001
Stress	18.58 ± 2.55	16.86 ± 2.42	5.84	<0.001
*Control group*				
Depression	17.89 ± 1.75	17.82 ± 1.98	0.19	NS
Anxiety	17.84 ± 2.06	17.76 ± 2.07	0.65	NS
Stress	18.33 ± 1.51	18.18 ± 1.56	0.78	NS

NS: not significant.

## Data Availability

All collected data were securely stored (i.e., in locked filing cabinets and password-protected computers), accessible only to the research team.

## Supplementary Materials

Supplementary Materials STROBE Statement: checklist of items that should be included in reports of *cross-sectional studies*.
